# Cancer

**Published:** 1858-05

**Authors:** 


### CANCER

Is dreaded, by women especially, more than any other disease, because it is always terribly painful, always slow in progress, to end in certain death—such, at least, is the general, educated medical sentiment. The ancients gave it two names, one meaning "marble," from its hardness at first; the other, "*Cancer*," signifying "Crab," from its ugly spraggling appearance. We believe that its extirpation *always* hastens death. It may attack any part of the body, but oftenest the breasts of women, and the under lip—seldom the upper; usually the result of a bruise or the healing of a cut or sore, *with a hard scar.* Hence, all persons who have piles or fistula, are liable to have this fearful disease located in the lower bowel, called "*Rectum.*" The more vigorous the constitution, the more terribly will the person suffer, from the power with which such a constitution resists death.

It was *Cancer of the Rectum* which destroyed Mr. BENTON; and a melancholy interest will always be attached to the last volume of his "*Congressional Abridgment*"—from the fact that he worked at it until the very last day of his life, almost; and must have often and long worked in agony, which none but himself ever knew, because his stern and self-reliant nature would not bend to the rehearsal of personal sufferings.

In one sense of the word, only cancer could have killed the great statesman for a decade or two to come, because a youth of personal activity, and a long life of the strictest temperance in eating and drinking, had made him like a pine-knot. But as pain and agony always flee the dying couch of slow disease, so the great Missourian's last connected words were,

"COMFORTABLE AND CONTENT,"

indicative of freedom from suffering. The question, what causes *Rectal Cancer?* comes with great force, under the circumstances. When persons fail to have a daily action of the bowels, the first step is taken towards piles, these lead to fistula of the parts, a dangerous, troublesome, and disgusting malady, often incurable, and when cured, leaving a *hard scar*—as is also the case with piles, or any other healed sore; but when this hard scar is in a place of great flexibility or mobility, where the muscles are always dragging on one another—as in the lip or rectum—or where there are glands—as in the breast—cancer often follows. The very moment, therefore, that a person notices that a day often passes in which the bowels do not act, he does thenceforth become daily more liable to these ailments; and will most certainly have some one of them, if the habit is not broken up, or if some more specially fatal disease does not, in the mean time, set in.

The frequent taking of any medicine to remove costiveness, does, at the most, only protract the event—making it, however, the more certain. A resort to injections—to say nothing of its filthiness and trouble—is no better than the daily use of Turkey rhubarb root, or patent pills. The only safe plan of removing the habit of constipation is, by means of the daily food; the general principles of which are laid down in our book on "Consumption," page 186, under the heading of "*Rules for Health*," sent by us, post paid, for one dollar.

